# Influence of Crystalline Admixtures on the Short-Term Behaviour of Mortars Exposed to Sulphuric Acid

**DOI:** 10.3390/ma12010082

**Published:** 2018-12-27

**Authors:** Victoria Eugenia García-Vera, Antonio José Tenza-Abril, José Miguel Saval, Marcos Lanzón

**Affiliations:** 1 Departamento de Arquitectura y Tecnología de la Edificación, Universidad Politécnica de Cartagena, 30203 Murcia, Spain; victoria.eugenia@upct.es (V.E.G.-V.); marcos.lanzon@upct.es (M.L.); 2 Department of Civil Engineering, University of Alicante, 03690 Alicante, Spain; jm.saval@ua.es

**Keywords:** crystalline admixture, chemical exposure, sulphuric acid attack, durability, Xypex

## Abstract

Using durable materials is a sustainable solution for extending the lifetime of constructions. The use of crystalline admixtures makes cementitious materials more durable. They plug pores, capillary tracts and microcracks, blocking the entrance of water due to the formation of crystals that prevent the penetration of liquids. The literature has covered the performance of these admixtures on concrete, but studies on mortars are still scarce. The aim of this study is to investigate the effect of an aggressive environment (sulphuric acid solution—3 wt%) on mortars produced with different percentages of a crystalline admixture (1%, 1.5% and 2% by weight of cement content). Physical and mechanical properties were studied after immersing the mortars in a H_2_SO_4_ solution for 90 days. It was found that, after a 90-day sulphuric acid exposure, mortars with the crystalline admixture showed greater compressive strength than the control mortar, besides exhibiting lower mass loss. However, the crystalline admixture did not produce any significant effect on the capillary water absorption coefficient. In a nonaggressive environment, and in the short term, the crystalline admixture did not have a significant effect on the compressive strength, the capillary water absorption coefficient or the ultrasonic pulse velocity.

## 1. Introduction

Nowadays, cement-based materials are exposed to acids because of environmental pollution, contact with some soils, ground waters, industrial waste-waters or sewers. These conditions can be found in some foundations, sanitation networks, treatment plants, agricultural and farm facilities, etc. [1,2,3]. Materials that are designed to work in aggressive chemical conditions must be durable materials, that is, materials with the capacity to withstand, for its service life, the physical and chemical conditions to which they are exposed [4]. Architects and engineers are vested with the responsibility of designing and building constructions by optimising the existing but limited resources in nature. Working with durable materials contributes to sustainability since they help to maintain the existing resources and avoid causing harm to the environment.

Concrete is susceptible to acid attack because of its alkaline nature. Sulphuric acid is particularly corrosive due to the sulphate ion in addition to the dissolution caused by the hydrogen ion causing the deterioration of cement-based materials. The reaction of the sulphuric acid (H_2_SO_4_) with the chemical compounds of cement hydration (calcium silicate hydrate, portlandite and calcium sulphoaluminate hydrate) generates gypsum, amorphous hydrous silica and aluminum sulphate. [3]. These chemical reactions, located in the regions close to the surface, provoke a profound degradation of the hydrated cement paste in terms of physical and mechanical aspects, such as compressive strength loss, mass variation, cracking, softening and decohesion. This process leads to spalling and exposure of the interior of the concrete structure [5,6].

To improve acid attack resistance in cement-based materials, the matrix must be dense, with low permeability, and it is advisable to use in the production a sulphate-resistant cement, high alumina cement, etc. [7,8,9]. Some authors state that effective strategies for manufacturing cementitious materials that are durable when exposed to acid attacks involve the following: (i) controlling the materials used during the manufacturing process; (ii) producing them with a low water/cement ratio; and (iii) guaranteeing suitable curing conditions [10,11].

A very popular strategy used to produce durable cement-based material is adding admixtures and additives [12,13,14,15]. Some studies utilise nanomaterials to reduce the porosity and water absorption of cement-based materials [16,17], which leads to enhanced durability and improved performance when these materials are exposed to acid attacks. Examples of such nanotechnologies are nano silica, nano alumina, titanium oxide, carbon nanotubes and polycarboxylates [18,19,20,21,22]. Water-repellent materials are also used to increase durability in acid attacks, since they reduce the affinity of capillary pore surfaces to moisture [23,24]. Examples of hydrophobic compounds used as admixtures are powdered stearates, oleates and products based on silanes and silicones [25,26,27]. When adequate recycling materials are available, another strategy to enhance the concrete durability is the use of aggregates derived from sanitary ceramics. Such concretes are characterised by low water absorption, higher mechanical strength and higher water-resistance compared to conventional concrete. Moreover, they have more resistance to chemically aggressive environments compared to conventional concretes [28,29,30,31].

Crystalline admixtures can also help increase the durability of cement-based materials, especially when cementitious materials are in acid environments. According to various manufacturers of products for crystalline waterproofings for concrete—Xypex, Kryton, Penetron, etc.—there are three ways of applying the waterproofing systems: (i) a coating applied as a cementitious slurry to the surface of existing concrete structures; (ii) an admixture added to the concrete at the time of batching; and (iii) a dry-shake to the fresh concrete surface. In all cases, the protection system is a blend of Portland cement, fine treated silica sand and undisclosed chemicals that are the intellectual property of the manufacturer. The catalytic reaction of the chemicals in the admixture occurs as long as there is moisture in the cement-based materials. These chemicals react with calcium hydroxide and other products resulting from cement hydration. The reaction generates nonsoluble crystalline formations that fill the pores and capillary tracks of cement-based materials. The crystalline structure becomes a permanent and integral part of the concrete matrix. Thus, the concrete becomes permanently sealed against the penetration of water or liquids from any direction [32].

The crystallisation process of these admixtures is not immediate, and the complete waterproofing effect is not achieved until approximately 12 days after the concrete creation [33], as long as there is enough moisture for chemical reactions to occur. Moreover, it is especially important to have a moist curing environment to obtain better performance [34]. There are several studies that focused on testing the waterproofing effect of these admixtures [33,35], as well as the impact of an aggressive medium on concretes made with crystalline-based waterproofing products [36,37].

However, it is still a challenge to find high durability mortars manufactured with these types of admixtures in aggressive environments. In this study, a crystalline protection product (applied as an admixture) was used to make mortars with different percentages of admixture (1 wt%, 1.5 wt% and 2 wt%). Their behaviour when they were exposed to an aggressive environment (sulphuric acid solution—3 wt%) was evaluated. Physical (mass loss, ultrasonic pulse velocity and capillary water absorption coefficient) and mechanical (compressive strength) properties were studied after the mortars were immersed in an H_2_SO_4_ solution for 28 and 90 days. Moreover, the textural alterations due to the acid attack were evaluated using microscopy.

## 2. Materials and Methods

### 2.1. Materials

**Figure 1:**
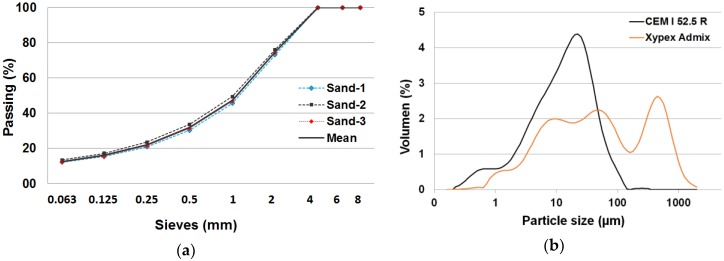
(**a**) Particle-size distribution of the limestone sand. (**b**) Particle-size distribution (done by laser granulometry) of the cement (CEM I 52.5 R) and the admixture (Xypex Admix).

The cement used for the manufacture of the mortars was a high early strength type belonging to the European cement class CEM I 52.5 R [38]. Limestone sand was used as a fine aggregate (D_i_/d_i_ = 0/4). The particle-size distribution was calculated according to the procedure of UNE-EN 933-1 standard [39]. The distribution was obtained for three samples of sand and the mean values were calculated (Figure 1a). Xypex Admix C-1000 NF (Xypex Chemical Corporation, Richmond, BC, Canada) was added in three different concentrations (1 wt%, 1.5 wt% and 2 wt% of the cement weight) depending on the kind of mortar. The specific surface areas of the cement and crystalline admixture (Xypex Admix) used were 1.48 and 0.642 g/m^2^, respectively (determined by the Brunauer–Emmett–Teller—BET—method), and their particle-size distributions are plotted in Figure 1b. In total, four types of mortars were made: (i) control mortar (A mortar); (ii) mortar with 1 wt% of Xypex (B Mortar); (iii) mortar with 1.5 wt% of Xypex (C Mortar); and (iv) mortar with 2% of Xypex (D Mortar). The dosage used for all mortars was 1 part cement, 0.65 parts water and 3 parts limestone sand.

#### Chemical–Physical Characterisation

The crystallographic phases present in the composition of the cement and the admixture (Xypex Admix C-1000 NF) were analysed with X-ray diffraction (XRD) using a Bruker D8-Advance diffractometer (Bruker Española S.A., Madrid, Spain) with mirror Goebel, and a generator of X-ray KRISTALLOFLEX K 760-80F (power: 3000W, voltage: 20–60 KV and current: 5–80 mA) equipped with a X-ray tube with copper anode. The spectra were registered with angles from 4° to 60° at 0.05° stepping intervals in Ө-Ө mode, and the X-ray tube was utilised at 40 kV and 40 mA. The diffraction patterns were evaluated with HighScore software (Malvern Panalytical, Madrid, Spain) and powder diffraction database PDF4+. Moreover, the chemical compositions of the binder and the admixture were analysed with X-ray fluorescence (XRF) using an X-ray sequential spectrometer PHILIPS MAGIX PRO (Philips Ibérica, Madrid, Spain) equipped with a rhodium X-ray tube and beryllium window. The spectrometer was controlled with the software package SuperQ (Malvern Panalytical, Madrid, Spain) that also stored the measurements and results.

**Figure 2:**
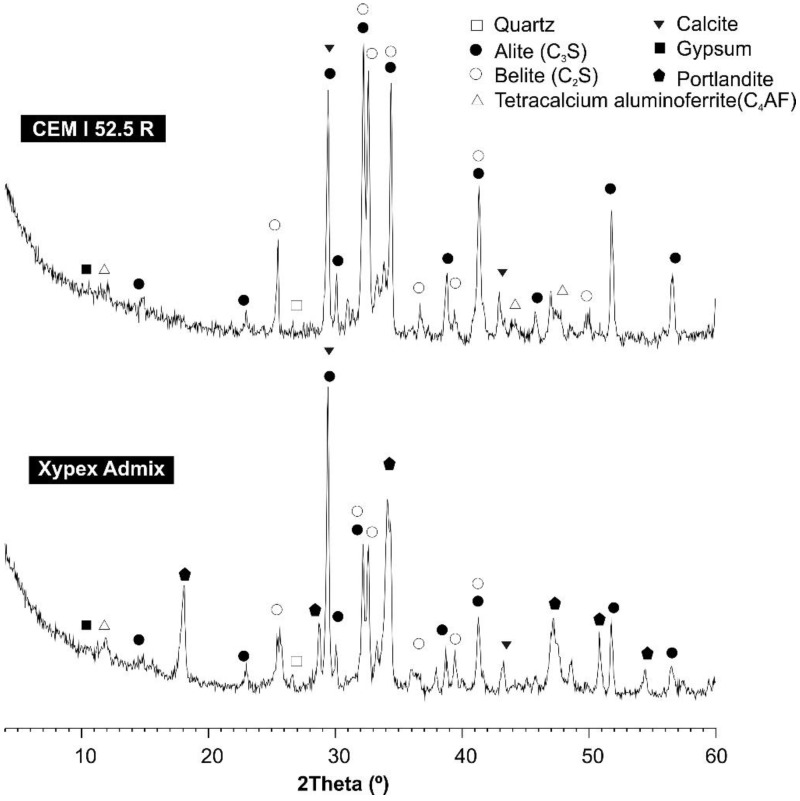
X-ray diffraction (XRD) spectra of cement type CEM I 52.5 R and Xypex Admix.

The XRF results (Table 1) show that both materials were mainly composed of CaO (cement: 55.4%; Xypex: 59.8%) and SiO_2_ (cement: 14.9%; Xypex: 8.1%), containing moderate proportions of Al_2_O_3_, SO_3_ and Fe_2_O_3_ (percentages between 1.9% and 4.6%). Only the cement contained moderate proportions of MgO (3.3%). The XRD spectra (Figure 2) revealed that both materials had common crystalline compounds, such as alite (C_3_S), belite (C_2_S), tetracalcium aluminoferrite (C_4_AF) and calcite (CaCO_3_). Gypsum was only detected in the cement, and portlandite and quartz were only detected in the Xypex admixture. The peak of portlandite in Xypex Admix may indicate that this admixture was partially constituted by hydrated cement.

### 2.2. Methods

#### 2.2.1. Manufacturing and Curing Process of the Mortars and Acid Attack Simulation

The different tests were performed on normalised mortar specimens—4 × 4 × 16 cm. The Xypex powder admixture was previously dry-mixed with the cement for 1 min with a laboratory mortar mixer. After that, the mortars were manufactured following the procedure of the standard UNE-EN 196-1 [40]. A total of 48 specimens were manufactured, 12 per each type of mortar (A, B, C and D). The specimens were cured in a humidity chamber for 28 days at 95% ± 2% of relative humidity and 20 ± 2 °C to complete the hardening process. After 28 days of curing, 24 of the specimens (representing half of the total of 48, that is, 6 specimens for each type of mortar) were exposed to a sulphuric acid solution for 90 days, and the other 24 were immersed in water for the same amount of time for reference purposes.

There are no European standards to test the chemical resistance of cement-based materials. However, Sokolowska et al. [41] studied the tests on cement-based materials according to ASTM (American Society for Testing and Materials) standards and they concluded that there is a lack of clear criteria for the evaluation of research results via ASTM methods. Due to the absence of standardised tests, in this study, the sulphuric acid attack was performed by immersing the specimens into an H_2_SO_4_ solution (3% w/w) in hermetically closed containers. This procedure has been used in previous studies to analyse the effect of acidic environments on cement-based materials [23,24]. A high concentration of sulphuric acid was chosen in order to accelerate their effects on the mortars and obtain the same degradation in less exposure time [12]. The performance of mortars against the acid attack was evaluated taking into account common parameters used in the literature for this purpose, such as mass variation and mechanical properties decrease [41]. The ultrasonic pulse velocity and the capillary water absorption were also studied [24]. The volume of solution was approximately four times the volume of the samples, as suggested by the ASTM C 1012-04 standard [42]. The H_2_SO_4_ solution was replaced weekly for a new solution (3% w/w) so that the concentration of sulphuric acid had minimal variation. After removing the specimens from the acid solution, they were brushed under a flow of water in order to remove the superficial layer of adhered material. After that, the specimens were introduced into a new solution. Once the acid exposure was finished, the specimens were dried at 105 ± 2 °C for 24 h, and then they were kept for 1 h at laboratory conditions before continuing with the tests.

#### 2.2.2. Scanning Electron Microscopy (SEM) Examination

The microstructural changes on the mortars, due to the action of the crystalline admixture and the effect of the sulphuric acid attack, were examined with scanning electron microscopy (SEM, Hitachi High-Technologies Canada, Inc., Toronto, ON, Canada) (Hitachi S3000N). Before the examination, small fragments from the mortar surfaces were removed, then softly dried at 60 °C for 24 h and finally metallised with Au–Pd (30 nm) in order to improve the image quality. The images were taken with the following conditions: secondary electrons mode, ultrahigh vacuum, 15 kV of accelerating voltage and variable working distance.

#### 2.2.3. Physical and Mechanical Properties of the Mortars after the Sulphuric Acid Attack

The impact of a sulphuric acid attack on cementitious material can be studied by evaluating its mass loss over the acid exposure time [43]. In this work, the mass loss was studied in seven stages of the acid simulation. To do this, the samples were weighed at the following intervals: (i) after completing their curing (i.e. at 28 days after their manufacture and 0 days of acid attack (t_28(0)_)); (ii) at 7 days of acid exposure (t_35(7)_); (iii) at 14 days (t_42(14)_); (iv) at 21 days (t_49(21)_); (v) at 28 days (t_56(28)_); (vi) at 56 days (t_84(56)_); and (vii) at 90 days (t_118(90)_) of acid exposure. The percentages of mass loss of the mortars were calculated taking into account the initial weights.

Compressive strength is a characteristic of the cement-based materials commonly used in the literature for analysing their performance against a chemical attack [12,43,44]. The reference mortars (that were kept in a nonaggressive environment) and the mortars exposed to the sulphuric acid attack were tested at t_56(28)_ and t_118(90)_. The compressive strength tests were performed according to the UNE-EN 196-1 standard [40]. The conventional mortar testing machine used had a load cell of 20 T capacity and was operated at a speed of 2.4 kN/s until failure.

Ultrasonic pulse velocity is a parameter that can be correlated with the elasticity modulus and therefore provides information about the stiffness of the material [45]. The ultrasonic pulse velocity test was performed according to the UNE-EN 12504-4 standard [46]. A total of four determinations were made per sample and the mean value was adopted. The test consisted of measuring the propagation time of the ultrasonic waves when crossing the longest dimension of the specimen (160 mm). Contact transducers emitting ultrasonic pulses at 54 kHz were coupled to the end sides of the specimens using a coupling agent. The wave speed was obtained from the propagation time and the length of the sample.

The impact of the sulphuric acid attack on the mortars was also evaluated studying the capillary water absorption of the specimens. The tests were conducted following the UNE-EN 1015-18 standard [47] for all the mortars (nonattacked and attacked) at t_56(28)_ and t_118(90)_. According to the standard, the water absorption coefficient is the slope of the line that joins the points corresponding to 10 and 90 min in the curve, representing the mass variation of water absorbed per unit area as a function of the square root of time; that is, the coefficient was computed using the formula

(1) $$C=\frac{M_{2}-M_{1}}{A\left(t_{2}^{0.5}-t_{1}^{0.5}\right)}$$

where:

*C* is the capillary water absorption coefficient, kg/(m^2^∙min^0.5^);
*M*_1_ is the specimen mass after the immersion for 10 min, kg;
*M*_2_ is the specimen mass after the immersion for 90 min, kg;
*A* is the surface of the specimen face immersed in the water, m^2^;
*t*_2_ = 90 min;
*t*_1_ = 10 min.

## 3. Results and Discussion

### 3.1. Compressive Strength

**Figure 3:**
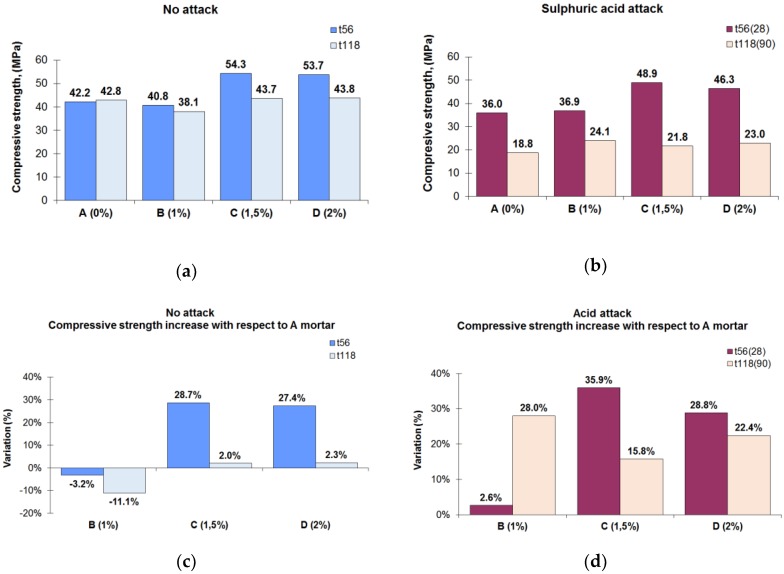
(**a**) Compressive strength in a nonaggressive environment at t_56_ and t_118_. (**b**) Compressive strength in an aggressive environment (sulphuric acid exposure) at t_56(28)_ and t_118(90)_. (**c**) Compressive strength differences of the mortars with the crystalline admixture with respect to the reference mortar in a nonaggressive environment. (**d**) Compressive strength differences of the mortars with the crystalline admixture with respect to the reference mortar exposed to sulphuric acid.

The results of the compressive strength tests showed that for a nonaggressive environment (Figure 3a) at t_56_ (56 days from the manufacture of the mortars), the mortars with the highest compressive strength were the C (54.3 MPa) and D (53.7 MPa) types compared with the compressive strength of the A (42.2 MPa) and B (40.8 MPa) mortars. Besides the obvious advantages in the use of the admixtures for mortars, there were drawbacks to take into account. One of those was the lowered final compressive strength compared with nonmodified concretes and mortars using some types of admixtures [48]. In the interval from t_56_ to t_118_, the only mortar that increased its compressive strength was the reference mortar (A mortar, without crystalline admixture), from 42.2 to 42.8 MPa, whereas in that same period, the three mortars with the crystalline admixture decreased their compressive strength. Usually, admixtures with an accelerated setting effect can reduce the strength of the concrete at later ages. In the high-strength mortars, a mechanism of deterioration of the hardened cement paste phase at the microscopic scale seems to lead to reduced strength at long ages [49]. The C and D mortars clearly had more compressive strength than the reference mortar at t_56_ (B mortar had 28.7% more compressive strength than the A and C mortars (27.7%)), although at t_118_, this difference was smaller (C mortar: 2%, D mortar: 2.3%) (Figure 3c). It was observed that the C and D mortars exhibited similar compressive strength to the reference mortar at t_118_, which was in accordance with previous research [33], where the crystalline admixture does not significantly affect the compressive strength of the concretes studied. However, the compressive strength of the B mortar was lower than that of the reference mortar, both at t_56_ and t_118_.

The compressive strength of the mortars exposed to 28 days of acid attack (56 days after manufacture) followed a pattern similar to that of mortars without acid attack (Figure 3b). The mortars with the highest compressive strength were types C (48.9 MPa) and D (46.3 MPa), and the lowest were types A (36.0 MPa) and B (36.9 MPa). The compressive strength at t_56(28)_ decreased in the same way for all mortar types (due to the acid attack), given as a reference the compressive strength without acid attack, for each mortar with the same curing time (t_56_). The reduction in compressive strength was a direct effect of the acid attack due to the microcracking caused by the formation of expansive compounds [50,51]. As expected, the compressive strength of mortar type B slightly exceeded the compressive strength of mortar A. When increasing the exposure time of the attack to 90 days (t_118(90)_), the compressive strength of all mortars decreased due to the effects of the attack. In this case, mortars with the admixture (B, C and D mortars) clearly had higher compressive strength compared with the reference mortar (B mortar had 28% more strength than mortars A, C (15.8%) and D (28.8%)) (Figure 3d).

On the other hand, a behavioural change in the compressive strength of the mortars was observed for 90 days of acid exposure. B mortar had the highest compressive strength, whereas for a 28-day exposure, the one with the highest compressive strength was C mortar.

### 3.2. Mass Loss Due to the Sulphuric Acid Attack

**Figure 4:**
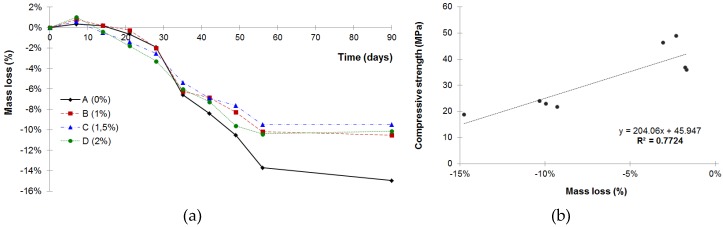
(**a**) Mass loss due to the sulphuric acid attack. (**b**) Correlation between compressive strength (MPa) and mass loss (%) after the acid attack.

The results of the mass loss test due to the sulphuric acid exposure (Figure 4a) showed that an acid attack caused a mass loss in mortars so that, as the acid attack continued longer, the mass loss of the mortars was higher. The compressive strength loss described above and the increment in mass loss when the exposure time increased were consistent with the results obtained in previous studies [43]. Moreover, a linear correlation (*R*^2^ = 0.7724) between the decrease in compressive strength and the mass loss was found (Figure 4b). Progression of the acid attack front caused an increase in porosity and permeability, leading to mass and strength loss [52]. Nevertheless, the mortars with the crystalline admixture (B, C and D mortars) behaved clearly better than the mortars without this admixture (A mortar) since they presented lower mass loss (B mortar: 10.5%; C mortar: 9.5 %; D mortar: 10.1%) than the mortar without the admixture (A mortar: 15%).

**Figure 5:**
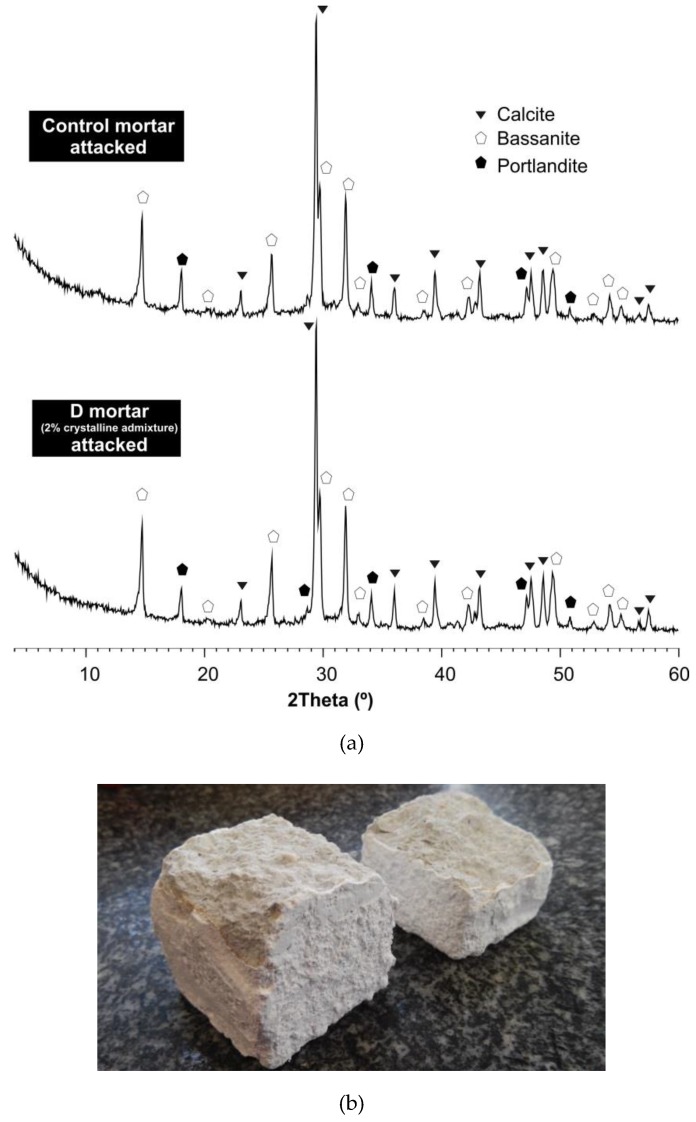
(**a**) X-ray diffraction spectra of control mortar and D mortar (with crystalline admixture) after 90 days of sulphuric acid attack; (**b**) Specimens exposed to the acid attack after compressive strength testing at t_118(90)_, where the massive formation of gypsum in the surface of the specimen can be seen.

As a result of the sulphuric acid attack, calcium sulphate (gypsum) was formed by the reaction of the acid with the calcium hydroxide (chemical reaction 2) and calcium silicate hydrate (chemical reaction 3) that were present in the hydrated Portland cement [3,5] and limestone sand (chemical reaction 4) [45,53]. The gypsum coating could also be observed with the naked eye (Figure 5b). The formation of gypsum after a sulphuric acid attack has been confirmed in the literature with XRD studies [52,54]. The chemical reactions produced by the acid attack resulted in a profound degradation of the hydrated cement paste, associated with a loss of compressive strength. When the concrete surface in addition to the acid attack was exposed to flowing water, the products of the degradation were carried away to a significant degree, causing a mass loss. Generally, an attack by free sulphuric acid is more severe than any with a neutral sulphate solution [3]. As mentioned in Section 2.2.1, the specimens were brushed and cleaned weekly to remove the gypsum formed on the specimen surface. The mass loss shown in Figure 4a was mainly associated with the amount of gypsum removed from the mortar surfaces during the brushing process. However, as the literature states [54], at the beginning of an acid attack, there is an increase in mass, which can be explained by the generation of gypsum in the pores and the cement–aggregate interface. This gypsum was difficult to remove even though the surface of the samples was brushed; therefore, at the first stage of the attack, a mass increase was found. To corroborate the existence of gypsum on the surface of the attacked mortars, an XRD analysis was performed. The samples were taken from fragments obtained from the surface of the specimens (4 × 4 × 16 cm) that had been broken in the compressive tests. The XRD analysis showed the presence of bassanite (CaSO_4_∙1/2H_2_O), which was the result of the thermal decomposition of gypsum, and it happened at 110 °C (chemical reaction 5) [55]. Therefore, it was consistent to find bassanite (hemihydrate phase of gypsum) after having exposed the specimens at 105 ± 2 °C continuously for 24 h. This result confirmed the previous existence of gypsum.

Ca(OH)_2_ + H_2_SO_4_ → CaSO_4_∙2H_2_O (2)

*x*CaO∙SiO_2_∙aq + *x*H_2_SO_4_ + *x*H_2_O → *x*CaSO_4_∙2H_2_O + SiO_2_∙aq (3)

CaCO_3_ + H_2_SO_4_ + 2H_2_O → CaSO_4_∙2H_2_O + CO_2_ + H_2_O (4)

CaSO_4_∙2H_2_O → CaSO_4_∙1/2H_2_O + 3/2H_2_O (5)

### 3.3. Ultrasonic Pulse Velocity

**Figure 6:**
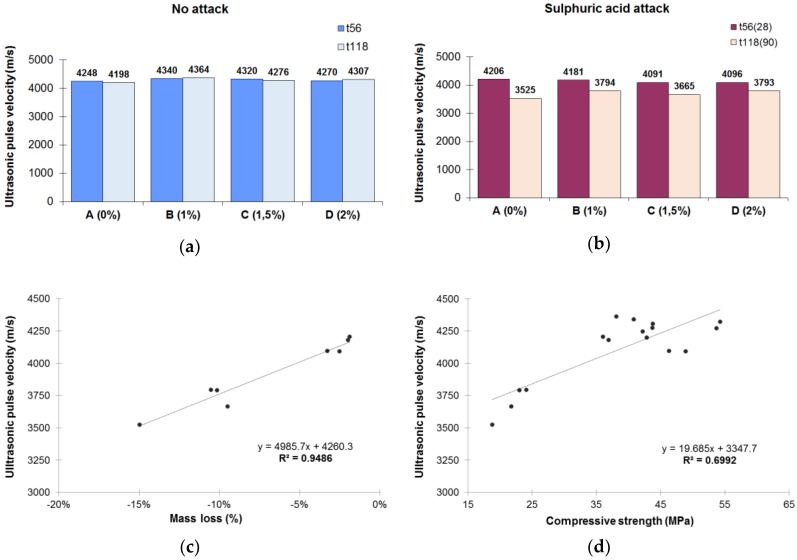
(**a**) Ultrasonic pulse velocity of mortars kept in a nonaggressive environment at t_56_ and t_118_. (**b**) Ultrasonic pulse velocity of mortars exposed to a sulphuric acid solution for 28 and 90 days. (**c**) Correlation between ultrasonic pulse velocity (m/s) and mass loss (%) after the acid attack. (**d**) Correlation between ultrasonic pulse velocity (m/s) and compressive strength (MPa).

Figure 6 shows the results of the ultrasonic pulse velocity test. This test consisted of obtaining the velocity (m/s) that the ultrasonic waves needed to cross the longest length of the specimen. The test was performed for mortars maintained in nonaggressive conditions (Figure 6a) and the ultrasonic velocity obtained was similar for all mortars in both t_56_ (from 4248 to 4340 m/s) and t_118_ (from 4198 to 4364 m/s). For mortars exposed to acid attack (Figure 6b), the ultrasonic velocity decreased for all mortars with respect the velocity obtained for a nonaggressive environment for both t_56(28)_ (from 4091 to 4206 m/s) and t_118(90)_ (from 3525 to 3794 m/s). To obtain a measure of the effect of the acid attack on the propagation velocity, the difference between ultrasonic pulse velocity at t_118_ for the mortars with and without attack was calculated. The measurements were taken after 90 days of acid attack, which is an action time long enough for the effects to manifest. It was found that the mortars with the admixture had less velocity decrease (B mortar: 13.0%, C mortar: 14.3% and D mortar: 11.9%) than the reference mortar (16.0%). In addition, the longer the acid exposure time, the lower the ultrasonic pulse velocity. The results demonstrate that the ultrasonic technique reflects the deterioration caused by sulphuric acid attack, providing information about the internal condition and quality of the mortars [56,57]. This is due to the large amount of cracks in specimens [58]. Indeed, in this work, two relationships between the involved variables were found: (i) correlation between the decrease in ultrasonic pulse velocity and the mass loss, and (ii) correlation between the ultrasonic pulse velocity and the compressive strength (Figure 6c,d). Recent studies have demonstrated [59,60] that the variation of the ultrasonic pulse velocity indicates a variation in Young’s modulus. Lower speed means lower Young’s modulus and greater deterioration of mortars.

### 3.4. Capillary Water Absorption Coefficient

**Figure 7:**
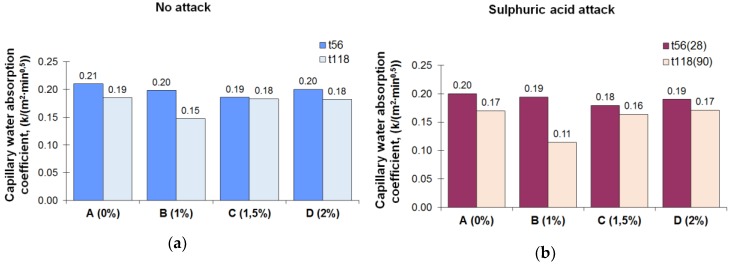
(**a**) Capillary water absorption coefficients of mortars kept in a nonaggressive environment at t_56_ and t_118_. (**b**) Capillary water absorption coefficients of mortars exposed to a sulphuric acid solution for 28 and 90 days.

The results of the capillary water absorption test are shown in Figure 7. For nonaggressive conditions and early ages (56 days from its manufacture), the absorption coefficient of the mortars with the crystalline admixture was slightly lower (B mortar: 0.20; C mortar: 0.19; D mortar: 0.20) than the absorption coefficient of the reference mortar (A mortar: 0.21). At t_118_, the absorption coefficients for all the mortars reduced but without reaching the values obtained for a water-repellent mortar [25]. These results agree with other studies which found that concretes treated with crystalline materials had slightly lower depth of pressure water penetration than the reference concrete in the short term [36,61] and negligible effects in terms of water vapor permeability [33]. In a sulphuric acid exposure, all mortars slightly reduced their capillary coefficients when compared to the values obtained for those same times in a nonaggressive environment. This reduction could be due to the formation of a gypsum coating on the surface so that the gypsum crystals obstructed the capillary net, reducing the absorption coefficient [24].

### 3.5. Scanning Electron Microscopy

**Figure 8:**
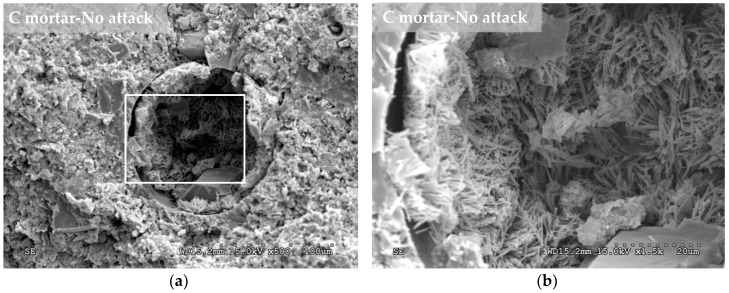

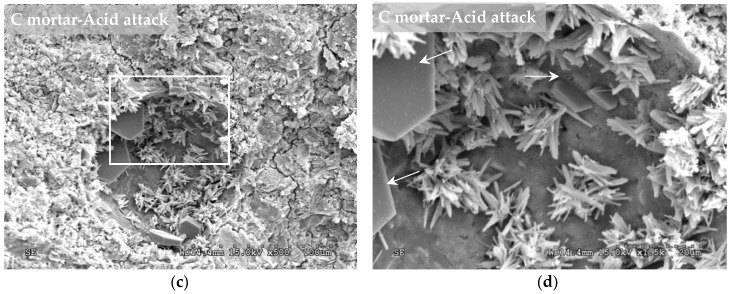
Scanning electron microscopy (SEM) images performed at 84 days (12 weeks) from the mortar manufacture. Left (**a**,**c**): images at 500× magnification. Right (**b**,**d**): zoom at 1500× magnification on the left image square. Top (**a**,**b**): mortar type C (with 1.5% of crystalline admixture) kept in a nonaggressive environment. Bottom (**c**,**d**): mortar type C exposed to a sulphuric acid attack for 56 days (8 weeks).

**Figure 9:**
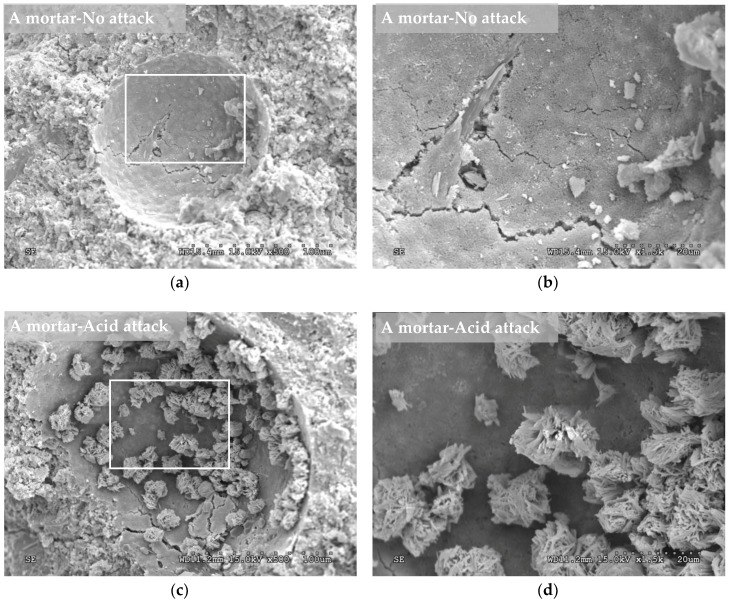
SEM images performed at 84 days (12 weeks) from the mortar manufacture. Left (**a**,**c**): images at 500× magnification. Right (**b**,**d**): zoom at 1500× magnification on the left image square. Top (**a**,**b**): mortar type A (0% of crystalline admixture) kept in a nonaggressive environment. Bottom (**c**,**d**): mortar type A exposed to a sulphuric acid attack for 56 days (8 weeks).

In the SEM images, crystalline structures were observed in the pores of the nonattacked mortars containing the crystalline admixture (Figure 8a,b). These structures had very similar shapes (needlelike crystal) to those that the admixture manufacturer assures are formed when the crystalline chemical reacts with the calcium hydroxide and other by-products of cement hydration. These crystalline formations are insoluble in water and fill and plug pores, capillaries and microcracks of the cementitious materials [62]. In addition, in the mortar manufactured with the Xypex admixture, the typical hexagonal shapes of the portlandite were observed (arrows in Figure 8d). Figure 8c,d shows images of the mortar with the crystalline admixture after being exposed to the sulphuric acid solution for 56 days (8 weeks). It is also possible to observe crystalline formations in the pore but with lower density than for the case of the nonattacked C mortar. The decrease in crystal density may be due to the acid attack that could be reducing the crystalline formations. In the SEM images corresponding to the nonattacked control mortar (Figure 9a,b), the needlelike crystalline shapes described above did not appear. After the acid attack, crystalline forms appeared in the control mortar (Figure 9c,d), which were different from those observed in the mortar with the admixture, and they could correspond to bassanite (hemihydrate phase of gypsum). The bassanite was the result of the effect of the sulphuric acid on the products of cement hydration and was detected in the diffractogram (Figure 5a).

## 4. Conclusions

This study analysed the effects of a sulphuric acid exposure on the physical and mechanical properties of mortars manufactured with a crystalline admixture in the short term (up to 90 days). The acid attack had significant consequences on the microstructure and the physical and mechanical properties of mortars. According to the results obtained in this study, the following can be concluded:

1. In a nonaggressive environment, the use of crystalline admixtures did not produce significant effects on the compressive strength of mortars. This is an expected result, since this type of admixture is not designed to increase the compressive strength of cement-based materials but their durability. After an acid attack, both the control mortar and the treated mortars experienced a decrease in their compressive strength. The longer the sulphuric acid exposure, the greater the compressive strength loss. However, mortars with the crystalline admixture showed greater compressive strength than the control mortar after 90 days of sulphuric acid attack. The compressive strength decrease, compared to the control mortar, was reduced between 16% and 28% depending on the amount of the admixture concentration.
2. Likewise, after a sulphuric acid attack, all the mortars (control and treated mortars) presented mass loss, being greater at longer exposure time. However, the treated mortars had lower mass loss after 90 days of sulphuric acid exposure. Their mass loss percentages were 9.5%–10.5% for the treated mortars and 15% for the control mortar.
3. In a nonaggressive environment, the crystalline admixture did not produce significant effects on the capillary water absorption coefficient of mortars, since it only produced a slight reduction of their absorption coefficients. Therefore, it should not be concluded that it is a hydrophobic material. After sulphuric acid exposure (90 days), all mortars reduced their absorption coefficients. In this case, the use of the crystalline admixture did not have a positive effect on the mortars as expected, since no significant differences were found between the control mortar and the treated ones. The reduction of the water absorption can be due to the formation of a gypsum coating that protects the surface of mortars. The coating of gypsum alters the superficial colour of mortars and can be appreciated with the naked eye.
4. The exposure to sulphuric acid had the effect of decreasing the ultrasonic pulse velocity, which implies a reduction of Young’s modulus of mortars and a probable loss of mechanical qualities. The decrease in velocity was greater when the exposure time increased. After 90 days of acid exposure, the mortars with the crystalline admixture showed a smaller decrease in the ultrasonic pulse velocity (between 11.9% and 14.3%) than the untreated mortar (16%) and thus a lower decrease of Young’s modulus.
5. SEM images corroborated the formation of insoluble crystalline products in the treated mortars. These formations had needlelike shapes and were located into the mortars’ pores.

This study has verified that the incorporation of crystalline admixtures improves the durability of mortars exposed to aggressive environments (sulphuric acid attack). Compared to normal mortars, crystalline admixtures added to mortars increase compressive strength and reduce mass loss. Consequently, a lower reduction of ultrasonic velocity is observed. Furthermore, incorporating crystalline admixtures in the mixing of the cementitious materials does not have the drawbacks of superficial treatments, such as the difficulty of adhesion with the substrate, the uneven thickness layer or the cost of applying the coating. Although, in the short term, the use of durable materials implies a higher initial cost without apparent benefit, in the long term, the decrease of maintenance and rehabilitation actions as well as the increase in the construction’s lifecycle is more cost efficient and, importantly, reduces the negative environmental impact.

## Figures and Tables

**Table 1:** X-ray fluorescence (XRF) characterisation of CEM I 52.5 R and Xypex Admix.

Oxides	CEM I 52.5 R (%)	Xypex Admix (%)
Na_2_O	0.23	1.20
MgO	3.29	0.82
Al_2_O_3_	3.36	1.98
SiO_2_	14.89	8.10
P_2_O_5_	0.14	0.06
SO_3_	4.62	2.09
Cl	0.11	0.03
K_2_O	1.06	0.44
CaO	55.36	59.77
TiO_2_	0.25	0.17
MnO	0.04	0.06
Fe_2_O_3_	3.06	2.08
SrO	0.12	0.06
Other elements	<0.3	<0.20
